# Samuel Johnson (circa 1769)

**DOI:** 10.3201/eid0806.020600

**Published:** 2002-06

**Authors:** Polyxeni Potter

**Affiliations:** * Centers for Disease Control and Prevention, Atlanta, Georgia, USA

**Figure:**
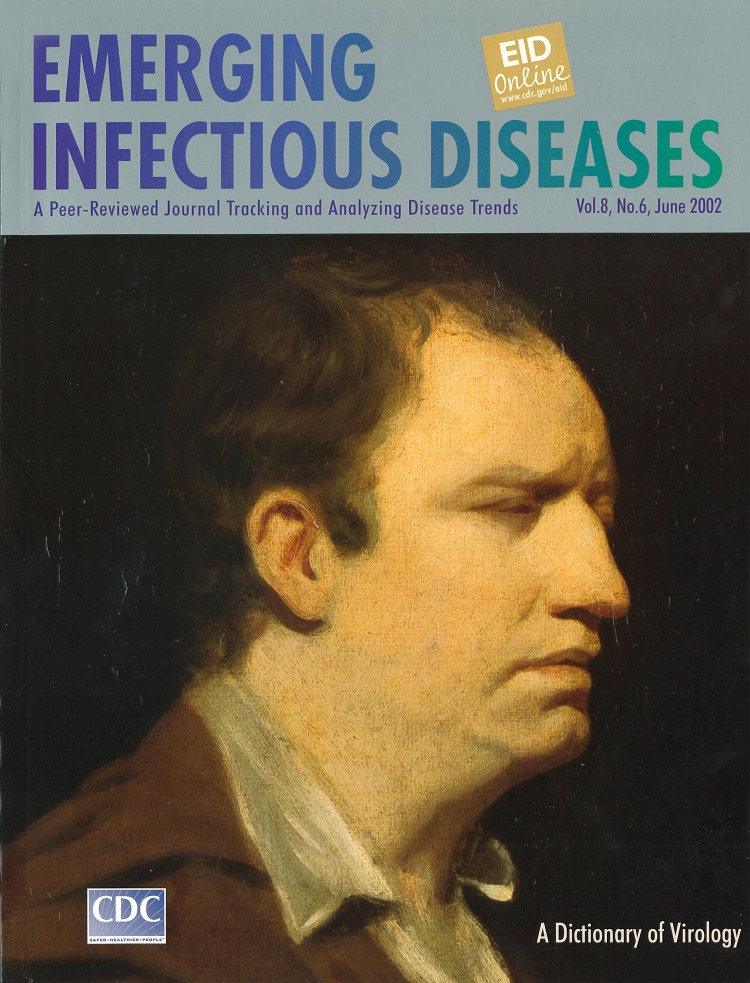
Samuel Johnson (circa 1769),reduced copy after Sir Joshua Reynolds By courtesy of the National Portrait Gallery, London.

**Samuel Johnson** was a poet, critic, lexicographer, and the author of the famous Dictionary, which he began in 1747. In 1755, Johnson's Dictionary appeared in two large folio volumes. It represented about 9 years of work and was written almost single handedly. Johnson had only the assistance of a few amanuenses to copy out the quotations he marked. Johnson originally approached Lord Chesterfield as a potential patron, but Chesterfield gave Johnson only a token sum (10 pounds). Thus, Johnson worked more or less unsupported, except by advances from booksellers. 

Although Johnson's financial situation was weak, his work remained without rival for almost 150 years, when the Oxford English Dictionary was created (1884–1928). For his dictionary, Johnson wrote the definitions of over 40,000 words, illustrating them with approximately 114,000 quotations drawn from every field of learning. 

Samuel Johnson was also a sitter in 16 portraits His circle of friends included Sir Joshua Reynolds, who painted Johnson in 1769. This portrait is known as the “Johnson Arguing” portrait. It illustrates Johnson’s mental concentration. Reynolds wrote that, when Johnson was excluded from conversation, “He remained but a few moments without speaking or listening. His mind appeared to be preying on itself; he fell into a reverie accompanied with strange antic gesticulations.”

Abstracted from: URL: http://newark.rutgers.edu/~jlynch/Johnson/Guide/dict.html, http://www.npg.org.uk/, and http://www.kirjasto.sci.fi/samuelj.htm

